# Quantitative design principles for biofunctional metal–organic frameworks: Stability thresholds, biointerface energetics, and therapeutic applications

**DOI:** 10.1016/j.mtbio.2026.103166

**Published:** 2026-04-28

**Authors:** Mousa Bohlooli, Mostafa Khajeh, Mansour Ghaffari-Moghaddam, Ali Pakdel

**Affiliations:** a Department of Cell and Molecular Sciences, Kharazmi University, Tehran, Iran; b Department of Chemistry, University of Zabol, Zabol, Iran

**Keywords:** Metal–organic frameworks, Coordination chemistry, Interaction–energy landscape, Biointerface energetics, Ligand exchange, Quantitative design principles

## Abstract

Metal–organic frameworks (MOFs) combine crystalline order with exceptional coordination-chemical tunability, enabling systematic control over metal–ligand bonding, lattice dynamics, and interfacial energetics. Although MOFs have been widely explored for biomedical applications—including drug delivery, imaging, and sensing—their behaviour under physiological conditions remains difficult to predict due to the lack of quantitatively defined, coordination-driven design principles. Consequently, most reported systems continue to be developed through empirical synthesis–evaluation cycles that incompletely sample the accessible chemical space. Across established MOF families, hydrolytic stability and biological performance vary by more than an order of magnitude and are governed primarily by coordination chemistry rather than framework topology alone. Zinc-based frameworks such as MOF-5 and ZIF-8 undergo rapid proton-assisted ligand displacement and framework collapse on the order of hours in phosphate-buffered saline, whereas zirconium-based UiO-66 and UiO-67 preserve their crystallographic integrity for several days under identical conditions. These contrasting behaviours correlate systematically with coordination-derived activation barriers, adsorption energetics governing biointerface formation, cargo–framework binding strengths, and surface-charge-dependent uptake pathways. This review introduces a semi-quantitative Interaction–Energy Landscape (IEL) framework that links molecular-level coordination energetics to experimentally reported trends in hydrolytic and structural stability, biointerface evolution, and functional performance. By consolidating recurring coordination-derived energetic constraints across biofunctional MOFs, the IEL framework defines a transferable design envelope connecting coordination chemistry with emergent biological function, showing how adaptive biofunctionality arises from deliberately constrained interaction-energy landscapes rather than intrinsic material “intelligence”.

## Highlights

• Introduces a semi‑quantitative energy‑landscape method linking coordination energetics to MOF bioactivity.
• Shows coordination‑derived energy barriers control hydrolytic stability and bio-interface response.
• Demonstrates that protein corona formation alters interfacial energetics and biological interactions.
• Defines energetic thresholds for stimuli‑responsive cargo release without invoking intrinsic material “intelligence”.
• Identifies surface chemistry and post‑synthetic modification as key factors governing biological stability in MOFs.

## Graphical abstract

**Figure:**
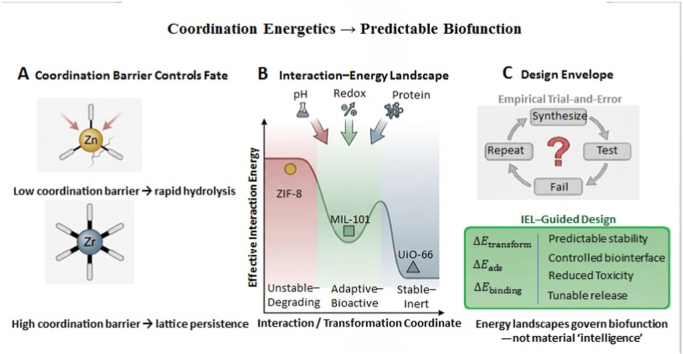

## 1 Introduction

Metal–organic frameworks (MOFs) have attracted sustained attention as modular coordination architectures whose structural regularity, high internal surface areas, and chemically tunable metal–ligand environments allow systematic control over lattice energetics and reactivity under aqueous and physiological conditions [1,2]. In contrast to conventional nanocarriers—whose functional behaviour often remains largely invariant despite fluctuations in pH, redox gradients, enzymatic activity, and metabolite composition—MOFs translate these environmental perturbations into measurable changes in coordination strength, energetic barriers, and pore accessibility. This capacity to encode external stimuli into adjustable coordination-energetic landscapes underpins their emerging use as responsive platforms for therapeutic modulation, rather than passive carriers [3,4].

In the context of MOF biointerfaces, the term stability encompasses several related but distinct phenomena. In this review, we focus on three biointerface-relevant forms of stability: hydrolytic stability (resistance of the framework to water-mediated degradation under physiological conditions), colloidal stability (the ability of MOF particles to remain dispersed in biological media without aggregation), and structural stability in biological environments (persistence of the framework architecture during exposure to physiological conditions).

Zirconium-based frameworks such as UiO-66 and UiO-67 exemplify hydrolytic robustness arising from highly connected Zr_6_O_4_(OH)_4_ clusters, maintaining pore topology under physiological conditions [5]. Conversely, low-connectivity systems including MOF-5 and ZIF-8 undergo rapid protonation-driven degradation, highlighting the need for quantitative energetic descriptors—${\Delta G}_{barrier\;}$, ${\Delta G}_{\text{interaction}\;}$ and ${\Delta G}_{\text{surface}\;}$—to predict biological lifetime and functional persistence [6,7]. Upon entry into biological fluids, however, the effective biological identity of nanoscale materials—including MOFs—is rapidly reshaped by protein corona formation, which strongly influences colloidal stability, cellular uptake pathways, and immune recognition. Studies on functionalized MOF nanoparticles demonstrate that subtle surface-chemical modifications can generate distinct and stabilizing corona compositions, producing measurable changes in aggregation behaviour and cellular interactions [8,9].

Stimuli-responsive behaviour further distinguishes MOFs from conventional carrier architectures by coupling framework dynamics directly to coordination-energetic perturbations. Flexible lattices such as MIL-88 undergo reversible changes in pore dimensions in response to variations in solvent composition or pH [10], while ZIF-8 exhibits acid-induced disassembly governed by proton-assisted weakening of metal–ligand interactions, a behaviour relevant to tumoral and endosomal environments [7]. Extensions of this concept in multi-stimuli-responsive systems emphasise threshold-dependent energetic modulation (ΔE), whereby multiple biological inputs collectively lower effective barriers to trigger structural transformation or cargo release, rather than invoking intrinsic logical processing. In parallel, machine-learning-guided design and high-throughput screening approaches have demonstrated high predictive performance across large MOF libraries, enabling systematic mapping of hydrolytic and structural stability, reactivity, and toxicity trends within defined chemical domains [11]. While these examples illustrate how external stimuli modulate MOF behaviour through changes in coordination strength and energetic barriers, they also underscore a key limitation of structure-centric descriptions. Framework composition, topology, or pore metrics alone cannot fully account for biological performance in dynamic media, where proteins, ions, and metabolites continuously reshape interfacial energetics. This recognition motivates a shift from static structural descriptors toward an interaction-energy-centred perspective capable of rationalising different forms of stability, transformation, and biological response across diverse MOF systems. Framing MOF–bio interactions in energetic terms therefore enables not only post hoc interpretation of experimental outcomes, but also prospective, energy-guided design and triage decisions prior to synthesis and in vivo evaluation. While energetic descriptors do not capture the full biological complexity, they provide a common quantitative axis across otherwise disconnected experimental observations.

Beyond delivery, many MOFs exhibit intrinsic catalytic and imaging capabilities. Fe-based MOFs have been widely reported to display catalase- and peroxidase-like activities relevant to oxidative microenvironments [12,13], while lanthanide-based coordination frameworks, including Eu^3+^-containing systems, exhibit long-lived luminescence suitable for sensing and bioresponsive imaging applications [[14], [15], [16]]. Nevertheless, lanthanide-based coordination systems frequently require protective or coordination-environment engineering strategies to maintain structural stability in biological media [15,17]. Toxicity remains a central translational barrier, exemplified by the degradation of ZIF-8 into Zn–imidazolate species with documented cytotoxic and immunomodulatory effects [7,[18], [19], [20]]. Predictive screening supported by DFT-derived interaction energies encoded within QSPR and machine-learning models now achieves >80 % accuracy in forecasting hydrolytic stability, reactivity, and toxicity [21], an increasingly relevant capability as MOF-based therapeutic systems are evaluated against Phase-I-like dose-escalation principles that define maximum tolerated or biologically active dose ranges [11,17,22,23].

Despite these advances, current understanding remains fragmented. Structural energetics, stability, protein-corona evolution, stimuli-responsive logic, catalytic activity, toxicity, and translational constraints are typically examined in isolation, leaving the design of biofunctional MOFs largely empirical and slow to converge. Critically, the absence of an integrative framework that connects molecular-scale energetic determinants with biological decision-making processes represents a fundamental barrier to systematic optimization and regulatory progression.

The central thesis of this review is that this disconnect arises from the lack of a shared energetic language. Accordingly, we establish a unified, energy-centred framework that links structural determinants, surface chemistry, emergent biointerface identity, interaction-energy landscapes, multi-stimuli logic behaviour, and degradation pathways with translational considerations. By grounding each domain in ΔE-based descriptors supported by experimental and computational evidence, and by mapping these energetic metrics onto physiological constraints, we provide a mechanistic basis for designing MOFs capable of adaptive, logic-governed behaviour in biological environments. While previous reviews have addressed isolated aspects of hydrolytic or colloidal stability, biointerface interactions, or stimuli responsiveness, none have integrated these dimensions into a coherent, predictive framework driven explicitly by energetic principles.

## 2 Structural foundations of biofunctional MOFs

The structural foundations of biofunctional metal–organic frameworks arise from the interplay of coordination architecture, lattice energetics, solvent-driven perturbations and post-synthetic programmability. Together, these variables determine whether a framework maintains structural and hydrolytic stability, undergoes controlled adaptation or experiences accelerated degradation under physiological conditions. Whereas early MOF design emphasised surface area and pore metrics, biological performance is instead governed by the lattice response to solvation, competitive ligand exchange and ionic fluctuations—factors that reshape the interaction-energy landscape at the biointerface. It is this energetic topology, rather than geometric regularity alone, that dictates uptake selectivity, catalytic behaviour, degradation kinetics and ultimately in vivo pharmacokinetics [24].

Intrinsic framework stability originates from metal–ligand bonding. High-valent Zr^4+^ and Hf^4+^ nodes form oxo-bridged clusters with high M − O bond dissociation energies and low susceptibility to nucleophilic substitution, explaining the robust behaviour of UiO-type frameworks across physiological pH and buffer conditions [1,25]. These architectures form the structurally stable regime of the classical stability triad and support long-circulating behaviour with predictable clearance patterns. In contrast, Fe^3+^- and Cr^3+^-based carboxylate frameworks populate a transitional regime where controlled hydrolysis enables pH-responsive disassembly and time-modulated release [26]. Zn^2+^-based systems such as ZIF-8 degrade rapidly through proton-catalysed linker cleavage, representing the “unstable” class [27]. Across these categories, validated by multiple independent in vitro and in vivo studies, the electronic structure of the metal node correlates mechanistically with hydrolytic lability, providing an energetically grounded framework for rational MOF selection in biomedical applications [28].

This triad is summarized in Table 1, which consolidates representative experimental degradation behaviours together with literature rationalized stability ranges derived from mechanistic and computational analyses across the reported stability spectrum. These ranges are intended to anchor the classical stability triad to experimentally familiar energetic scales, without implying universal.

**Table 1:** Stability regimes of biofunctional MOFs (literature rationalized ranges).

Stability Class	Representative Frameworks	Relative M–L Stability Domain^a^	Degradation Half Life in PBS (37 °C)^b^	Representative Biological Behaviour	References
Stable	UiO-66, UiO-67, Hf-UiO	High (>25 kcal mol^−1^)	>72 h	Hydrolysis-resistant frameworks with prolonged structural integrity and prolonged systemic persistence	[1,2,24,25]
Transitional	MIL-100(Fe), MIL-101(Cr)	Intermediate (≈15–25 kcal mol^−1^)	4–24 h	Controlled hydrolysis with environment dependent (pH-responsive) disassembly and time- modulated release	[20,26,29]
Unstable	MOF-5, ZIF-8	Low (<15 kcal mol^−1^)	<12 h	Rapid proton-assisted degradation leading to fast framework collapse and burst release	[6,7,17,18,27]

a Reported ranges reflect order of magnitude stability domains inferred from comparative hydrolytic degradation kinetics and DFT-supported mechanistic analyses across distinct MOF families, rather than transferable activation free energies or predictive energetic thresholds measured under uniform conditions.

b Degradation half-lives determined from in vitro dissolution studies in phosphate-buffered saline (PBS, pH 7.4, 37 °C) monitored by powder X-ray diffraction (PXRD), dynamic light scattering (DLS), or metal-ion release assays as reported in cited references.

**Fig. 1:**
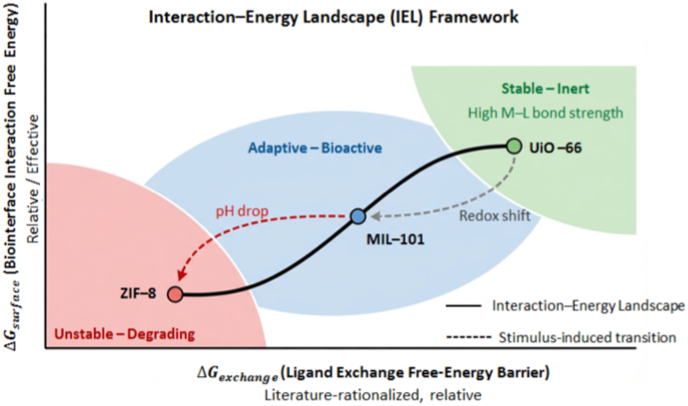
Interaction–Energy Landscape (IEL) framework for biofunctional metal–organic frameworks. Schematic representation of MOF behaviour as a function of the ligand exchange free energy barrier (${\Delta G}_{exchange}$) and the biointerface interaction free energy (${\Delta G}_{surface}$), which together define three characteristic functional regimes: a high-stability inert domain, an intermediate adaptive–bioactive domain, and a low-stability degradation-prone domain. The energetic ranges shown represent approximate, effective interaction energy domains synthesized from comparative experimental observations and literature-rationalized mechanistic analyses, rather than absolute thermodynamic quantities or predictive thresholds. Representative frameworks (UiO-66, MIL-101, and ZIF-8) are included for contextual illustration only. External stimuli, such as pH variation or redox fluctuations, are depicted as drivers that can shift frameworks across regions of the landscape by modulating ligand coordination stability or surface interaction energetics.

This stability triad can be embedded into a broader interaction–energy landscape that links ligand-exchange barriers and biointerface adsorption energetics to functional regimes (Fig. 1). In this framework, the ligand-exchange free-energy barrier (${\Delta G}_{exchange}$) describes the energetic cost associated with coordination rearrangement or ligand substitution at the metal node, while the biointerface interaction free energy (${\Delta G}_{surface}$) represents the effective adsorption or binding energetics governing MOF interactions with biomolecules and cellular interfaces [24]. Together, these descriptors provide a graphical basis for the interaction–energy landscape (IEL) framework developed throughout this review. Throughout this work, energetic descriptors are expressed as effective free-energy terms (ΔG) reflecting experimentally observed interaction trends under physiological conditions.

Practical deployment reflects these stability trends. Zr^4+^/Hf^4+^-based frameworks exhibit the highest structural stability and therefore remain intact for prolonged periods under physiological conditions. MIL-100 and MIL-101 display intermediate stability (t_1_/_2_ ≈ 6.8 ± 1.3 h), allowing controlled degradation in aqueous environments. In contrast, ZIF-8 lies at the labile end of the spectrum and readily decomposes under mildly acidic conditions [24,[26], [27], [28]]. When evaluated against physiological constraints—including ionic strength, protein corona evolution, and redox fluxes—these framework stability regimes provide a more mechanistically informative basis for assessing translational suitability than conventional topological descriptors such as connectivity or surface area.

Defective metal sites are recurrent degradation hot spots in Fe-based metal–organic frameworks, as coordinatively unsaturated environments exhibit enhanced electronic softness that lowers the activation barriers for hydrolytic attack. Post-synthetic modification (PSM) has therefore been investigated as a mitigation strategy to reduce water accessibility to these vulnerable regions. In Fe-based MIL-type frameworks, experimental evidence demonstrates that linker-level hydrophobic grafting can attenuate hydrolysis-driven degradation by sterically shielding the coordination environment from direct water contact and slowing structural breakdown under aqueous conditions [29]. Complementary approaches—including amine functionalization, partial linker exchange, and controlled defect modulation—enable fine adjustment of charge density, hydrophilicity, and biomolecular affinity, thereby expanding the available design space for tuning biointerfacial and colloidal stability and facilitating biological interfacing [28].

The UiO-66-NH_2_ family illustrates how linker environment influences node energetics. Amine substituents reshape hydrogen-bonding networks and adjust ${\Delta G}_{adsorption\;}$ for metabolites and proteins, resulting in tunable catalytic and recognition behaviour under physiological conditions [26,30,31]. Computational and machine-learning-assisted studies show that ligand-exchange kinetics and hydrolytic resilience are primarily determined by the electronic structure of the Zr_6_-oxo cluster rather than geometrical symmetry alone [30]. This principle extends across other architectures. In MOF-808, for instance, ΔG-driven ion-exchange trends among alkali metals correlate strongly with the node's electronic structure and can be used to predict selectivity and degradation pathways [30,32,33].

Beyond physicochemical stability in biological environments, structural modularity enables programmable functionality. Metal clusters can serve as redox-active or photoresponsive hubs, while organic linkers encode polarity, flexibility and functional-group presentation. Integrating responsive motifs transforms MOFs from static scaffolds into adaptive materials capable of sensing and responding to biochemical stimuli. Machine-learning-guided inverse-design frameworks now accelerate prediction of solvation behaviour, degradation energetics and interface stability across chemical space, facilitating rational development of MOFs that operate reliably in immune-modulated, redox-variable or enzyme-rich environments [30].

Collectively, these structural principles establish the mechanistic bridge between molecular design and biological performance. Biofunctional MOFs derive their biomedical relevance from the ways in which their architectures encode energetic logic, determining hydrolytic and structural stability, reactivity and responsiveness to physiological stimuli. Within the Interaction-Energy Landscape (IEL) framework, structural motifs gain biological significance only insofar as they generate specific energetic pathways. These foundations provide the basis for the biointerface analysis introduced in Section 3.

## 3 Biointerface and the Interaction–Energetics Landscape (IEL)

Biological behaviour and biointerface-dependent structural stability of metal–organic frameworks are defined at the interface where ordered lattices encounter proteins, ions, metabolites, membranes, and redox-active species. Structural descriptors alone fail to predict this behaviour, because biological recognition is governed primarily by energetic transitions rather than geometric regularity. The Interaction–Energetics Landscape (IEL) framework formalises this concept by decomposing interfacial processes into four principal energetic terms— ${\Delta G}_{approach}$, ${\Delta G}_{adsorption}$, ${\Delta G}_{exchange}$, and ${\Delta G}_{transform}$—which together determine adsorption, reorganization, ligand displacement, and transformation under biological conditions [34,35]. The IEL framework provides a semi-quantitative conceptual map of these biomolecule–MOF interactions. Computational approaches such as density functional theory (DFT) and machine learning (ML) complement this framework by offering atomistic or predictive insights that refine the interpretation of specific IEL energetic domains [11].

Protein corona formation represents the most immediate manifestation of effective adsorption free-energy (${\Delta G}_{adsorption\;}$) landscapes. Subtle variations in surface chemistry or defect density can reshape protein-binding preferences, yielding distinct corona fingerprints with major downstream biological consequences [8]. Empirical studies on UiO-66-NH_2_ and related Zr-based systems show that hydrogen-bonding and electrostatic contributions modulate early adsorption events and guide the evolution of the soft corona [8,36].

**Fig. 2:**
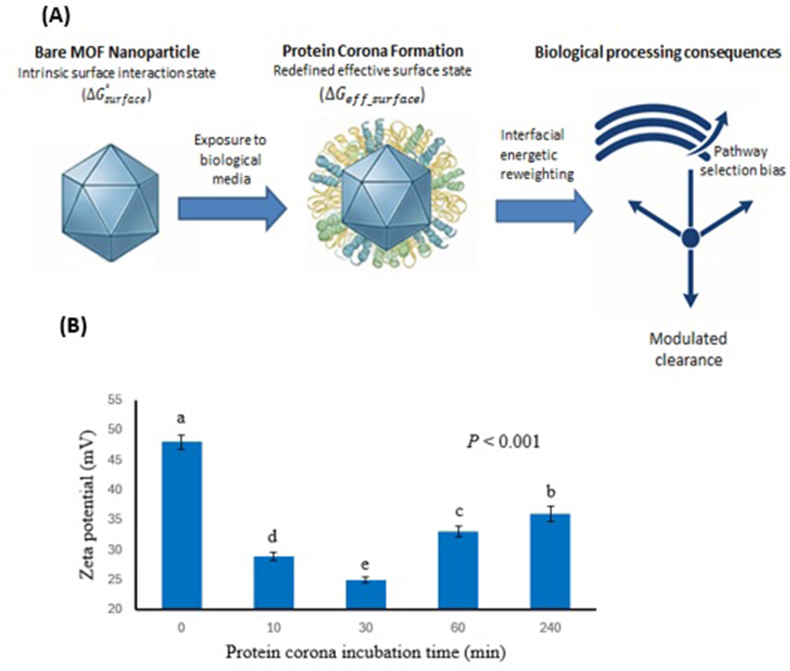
(A) Protein corona–driven redefinition of MOF surface interaction states within the Interaction–Energy Landscape (IEL). Adsorption and dynamic reorganization of serum proteins on initially bare MOF nanoparticles modify the effective surface interaction state (${\Delta G}_{surface}$, operational), producing energetic reweighting at the biointerface that biases—rather than strictly determines—downstream biological processing pathways, including cellular uptake and systemic clearance. The schematic is qualitative and illustrates generalized trends across MOF nanoparticles rather than a specific experimental system or quantitative free-energy profile. (B) Zeta potential of nanoparticles as a function of serum incubation time during protein corona formation. Reproduced from Ref. [37] under the Creative Commons CC BY 4.0 license.

As schematically illustrated in Fig. 2, this rapid evolution of the protein corona reshapes the effective bioidentity of MOF nanoparticles, with direct consequences for ζ-potential, colloidal stability, cellular uptake routes, and immune recognition [8,9].

Comparative experimental studies consistently place albumin and transferrin interactions with Zr-based nodes within moderate binding regimes (≈6–12 kcal mol^−1^), whereas Zn-based frameworks such as ZIF-8 occupy substantially weaker interaction regimes (typically < ∼10 kcal mol^−1^). These energetic trends are consistent with the rapid hydrolytic degradation widely reported for Zn-based MOFs in aqueous and physiological environments [[38], [39], [40]]. Soft-corona reorganization is associated with an additional energetic penalty on the order of a few kcal mol^−1^, consistent with the reported 30–50 min corona maturation times observed for MIL-100(Fe) and MIL-101(Cr) systems [41].

Ion exchange and solvation-driven transformations follow similar energetic logic. For MOF-808 and MIL-100(Fe), relative affinities for Na^+^, K^+^, Ca^2+^ and phosphate correlate with the electronic structure of the metal-oxo clusters and the associated ${\Delta G}_{exchange}$ landscape, as documented in experimental ion-exchange studies [[42], [43], [44]]. Ion-exchange and solvation-driven transformations are governed by similar coordination-energetic principles in aqueous environments. In Zr_6_-based frameworks such as MOF-808, experimental studies have shown that relative affinities for alkali and multivalent cations, as well as phosphate species, are dictated by the electronic structure and ligand lability of the metal-oxo clusters, defining an effective ${\Delta G}_{exchange\;}$ landscape. Related solvation-induced exchange and coordination dynamics have also been observed in Fe-based MIL-100 systems [45].

Cellular internalization is governed primarily by interfacial energetic balances rather than geometric descriptors alone. While particle size modulates uptake pathways, ζ-potential variations, hydration forces, and membrane-contact free-energy balances play dominant and recurring roles in governing internalization behaviour. Changes in nanoparticle surface charge directly regulate adhesion energetics and membrane wrapping, as established in model nanoparticle–cell interaction studies [46] and reinforced by recent analyses of surface-charge-driven biointerfaces [47].

Ligand-modified metal–organic frameworks (MOFs) commonly exhibit pronounced ζ-potential changes upon protein adsorption, which enhance membrane anchoring and increase cellular uptake. These effects arise primarily from surface-chemistry-dependent hydration and electrostatic interactions rather than particle size alone, and are consistently reported across experimental studies on MOF nanoparticles [48] as well as comprehensive mechanistic reviews of MOF–cell interactions [49].

In parallel, structural heterogeneities such as defect-rich or chemically anisotropic MOF surfaces can locally amplify interfacial energies, creating high-affinity adhesion sites that act as nucleation points for membrane contact and internalization. This behavior is increasingly recognized as a general feature of MOF nanoparticles, reflecting the sensitivity of cellular uptake to nanoscale surface energetics rather than idealized bulk properties [47,49].

The energetic regimes summarized in Table 2 integrate experimentally reported trends in protein corona formation, interfacial adsorption energetics, ion exchange, structural rearrangement, and degradation across Zr-, Fe-, Cr-, and Zn-based MOFs, rather than discrete measurements obtained under identical conditions, as documented in prior mechanistic and biointerface studies of MOF–biological interactions [[36], [37], [38], [39], [40], [41], [42], [43], [44], [45], [46], [47], [48]].

**Table 2:** Conceptual Interaction–Energy Landscape (IEL) energetic regimes governing MOF biointerfaces.

IEL Parameter	Indicative Free Energy Domain^a^ (ΔG, approximate, kcal mol^−1^)	Biophysical Interpretation	Representative MOF Families	References
${\Delta G}_{\text{approach}}$	Low to moderate positive barrier (≈+4 to +11)	Energetic barrier governing initial biomolecule approach to MOF surfaces, influenced by hydration shells and surface electrostatics	UiO-66-NH_2_, MIL-100(Fe), MIL-101(Cr)	[38,41,46,47]
${\Delta G}_{\text{adsorption}}$	Moderately favorable adsorption (≈−8 to −20)	Free-energy gain associated with protein adsorption driving biomolecular corona formation	Albumin–UiO-66, transferrin–Zr-MOFs, weakly bound coronas on ZIF-8	[[39], [40], [41]]
${\Delta G}_{\text{exchange}}$	Moderate activation barrier (≈+8 to +16)	Activation barrier governing ligand or ion displacement during pH- or ion-responsive interfacial transitions	MOF-808, MIL-101(Cr), MIL-100(Fe)	[36,42,44,45]
${\Delta G}_{\text{transformation}}$	Strong net driving force (≈−28 to −44)	Net driving force for framework rearrangement, degradation, or phase adaptation under biological stress	ZIF-8, defect-rich MIL-100(Fe), functionalized UiO-66 variants	[40,42,43,48]

a Relative energetic domains synthesized from comparative literature trends in protein corona formation (DLS, ITC, QCM-D), ion-exchange equilibria (ICP-MS, NMR), structural rearrangement (PXRD, EXAFS), and degradation kinetics in representative MOF systems, rather than absolute thermodynamic values measured under uniform conditions.

Surface engineering strategies further validate IEL predictions. Surface passivation of MIL-100(Fe) through biopolymer or grafted organic coatings has been shown to suppress premature hydrolytic degradation, improve colloidal stability in physiological media, and extend blood circulation times by reducing nonspecific biological recognition [50,51]. Similarly, surface and ligand-level chemical modification of nanoscale MOFs, including linker substitution and noncovalent surface engineering, moderates interactions with reactive biological species and metabolites, thereby lowering metabolic liability and improving chemical resilience under physiological conditions [52]. Collectively, these energetic considerations of the MOF biointerface enable more consistent qualitative rationalisation of hydrolytic and structural stability, cellular uptake, and in vivo retention than purely geometric descriptors such as porosity or surface area [50,53].

In this view, MOFs function not as passive scaffolds but as energy-responsive machines, where adsorption, reorganization, ligand exchange, and transformation correspond to predictable transitions along the IEL. This mechanistic clarity provides the conceptual bridge between the structural principles defined in Section 2 and the higher-level therapeutic logic explored in subsequent sections, supporting the design of MOFs with programmed recognition, selective cellular uptake, and stimulus-responsive behavior [[52], [53], [54], [55]].

Taken together, the analyses in Section 3 demonstrate that MOF biointeractions are governed by coupled adsorption, reorganization, ion-exchange, and transformation energetics rather than by isolated structural descriptors. The energetic regimes summarized in Table 2 therefore represent a synthesis of experimentally observed trends across diverse MOF families, rather than absolute thermodynamic quantities measured under uniform conditions. To avoid over-interpretation of these regimes and to clarify their intended use in material selection and design, it is essential to explicitly define the scope, limitations, and quantitative meaning of the Interaction–Energy Landscape (IEL) framework.

The semi-quantitative free-energy ranges used in the IEL framework are grounded in experimental observations reported across the MOF biointerface literature. The ${\Delta G}_{\text{approach}}$ regime (0–5 kJ/mol) reflects weak, long-range electrostatic and van der Waals interactions during initial biomolecule–MOF encounters. These interactions are typically inferred from colloidal stability measurements such as dynamic light scattering (DLS) and ζ-potential analysis, and supported by low-signal calorimetric measurements including isothermal titration calorimetry (ITC) [38,46,47,52].

The ${\Delta G}_{\text{adsorption}}$ regime (5–50 kJ/mol) captures moderate adsorption energies associated with protein corona formation and reversible biomolecular binding, as characterized by surface plasmon resonance (SPR), fluorescence quenching, and calorimetric techniques [8,9].

The ${\Delta G}_{exchange}$ regime (20–80 kJ/mol) is grounded in degradation and coordination-exchange kinetics under controlled ionic conditions, monitored by inductively coupled plasma mass spectrometry (ICP-MS) and powder X-ray diffraction (PXRD). Temperature-dependent dissolution analyses of ZIF-8 and related systems further report activation energies consistent with ligand or ion displacement processes [18,40].

The ${\Delta G}_{\text{transform}}$ regime (≥60 kJ/mol) is estimated from hydrolytic stability studies, framework degradation in culture media, and thermal stress tests, all of which require significantly higher energetic inputs associated with irreversible structural transitions [17,18,24,27].

Importantly, these ΔG ranges represent approximate, order-of-magnitude interaction regimes derived from diverse experimental techniques and multiple MOF chemistries, rather than sharply defined thermodynamic constants. Partial overlap between regimes is expected depending on MOF composition, local ionic environment, and biomolecular identity.

Accordingly, quantitative outputs from DFT calculations or ML-based screening can be contextualized within these IEL energetic domains, allowing computational predictions to be interpreted within the broader interaction-energy landscape.

## 4 Scope, limitations, and quantitative interpretation of the Interaction–Energy landscape (IEL)

The Interaction–Energy Landscape (IEL) introduced in Section 3 (Table 2) is a design-level, semi-quantitative framework that consolidates experimentally reported trends in protein corona formation, ion exchange, structural rearrangement, and degradation across representative MOF families [[37], [38], [39], [40], [41], [42], [43], [44], [45], [46], [47], [48]]. These indicative energetic regimes are derived from comparative literature data—including adsorption affinities, ζ-potential shifts, ion-exchange kinetics, and stability assay (primarily hydrolytic and colloidal)—rather than absolute thermodynamic measurements obtained under identical experimental conditions.

Within this scope, the IEL is intended to enable relative comparison and rational triage of biofunctional MOFs by mapping coordination-chemical features onto experimentally observed biological behaviours. It provides a unifying energetic language for interpreting hydrolytic and structural stability windows, interfacial adaptation, and functional persistence across chemically distinct frameworks, and is most effective when applied to comparative analysis of Zr-, Fe-, Cr-, and Zn-based systems discussed throughout Section 3.

The energetic values represented in the IEL should be interpreted as effective, operational descriptors that integrate multiple coupled contributions, including coordination strength, adsorption and displacement barriers, hydration effects, electrostatic screening, and degradation timescales. They are not universal constants and should not be treated as absolute predictive thresholds independent of experimental context. Variations in particle size, defect density, surface functionalization, and biological medium composition introduce heterogeneity that cannot be collapsed into a single energetic coordinate.

Accordingly, the IEL complements but does not replace detailed experimental characterisation or mechanistic modelling. Its quantitative interpretation is anchored to well-characterised reference systems, such as UiO-66, MIL-100(Fe), MIL-101(Cr), and ZIF-8, whose biointerface behaviours and degradation trends are consistently documented in the surveyed literature [[37], [38], [39], [40], [41], [42], [43], [44], [45], [46], [47], [48]]. Read in this operational sense, the IEL functions as a calibrated, semi-quantitative map that connects coordination chemistry to emergent biological responses, while remaining agnostic to specific therapeutic indications.

## 5 Drug delivery and therapeutic modulation

The drug delivery behavior of MOFs is often described as inherently “responsive,” implying that specific structural motifs can translate into predictable stimulus-triggered therapeutic release. In reality, release behaviour is predominantly governed by adsorption strength, exchange barriers, and framework relaxation processes, rather than ill-defined notions of intrinsic responsiveness [56,57]. Across experimentally observed and computationally rationalized interaction landscapes, polar therapeutics interacting with Zr-based nodes exhibit thermodynamically favoured binding consistent with classical Lewis acidity and established principles of coordination and adsorption thermodynamics [57,58]. Hydrophobic drugs confined within relatively shallow interaction basins in porous frameworks tend to desorb on biologically relevant timescales, which helps explain their premature leakage during circulation unless additional kinetic or interfacial barriers are deliberately engineered. In contrast, MIL-100(Fe) frameworks introduce an additional functional stabilization component associated with the redox-active nature of Fe^3+^ nodes, which modulates ligand exchange, degradation behaviour, and biomolecule interactions. This redox-influenced coordination environment results in slower cargo release profiles and prolonged intracellular retention compared with more redox-inert MOF systems, consistent with experimentally observed degradation trends, surface engineering strategies, and redox-modulated MOF–biomolecule interactions reported for iron-based nanocarriers [50,59,60].

**Fig. 3:**
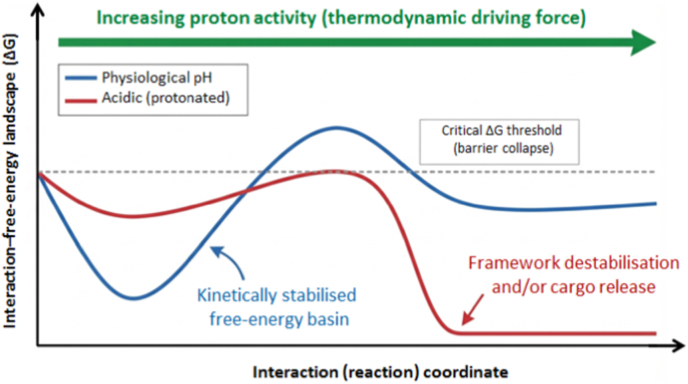
Protonation-induced free-energy descent in the interaction–energy landscape (IEL) of MOF–cargo systems. Under acidic conditions, increasing proton activity acts as an external thermodynamic driving force that lowers the free-energy barriers (${\Delta G}_{\text{barrier}}$), promoting framework destabilisation and rapid cargo release. This conceptual formalism is broadly applicable to other stimuli-driven transitions, as summarized in Table 3. The schematic illustrates qualitative, relative deformation of an effective (unitless) interaction free-energy landscape rather than quantitative potential-energy profiles.

Within an interaction–energy landscape (IEL) framework, variations in environmental acidity can be interpreted as an external thermodynamic field that reshapes the free-energy landscape governing MOF thermodynamic and chemical stability, transformation pathways, and biological persistence. Rather than exhibiting a single static energetic minimum, MOFs populate multiple metastable basins, enabling non-equilibrium transitions between ordered, disordered, and degraded states under physiological or pathological conditions [20,61]. Such behaviour, well documented for amorphous and glass-forming metal–organic networks, underscores that MOF hydrolytic stability in biomedical environments is fundamentally landscape-controlled rather than intrinsically fixed [20,61]. Under acidic or proton-rich conditions, linker protonation and coordination bond weakening reduce the energetic penalties associated with structural rearrangement, effectively deforming the local ΔG surface and lowering barriers that separate metastable basins from degradation or collapse regimes [[62], [63], [64]]. For imidazolate-based frameworks such as ZIF-8, experimental studies demonstrate that exposure to acidic aqueous environments induces facet-dependent destabilisation, consistent with heterogeneous landscape tilting rather than uniform dissolution [62]. First-principles calculations further confirm that increased proton activity weakens Zn–N coordination, promoting transitions from metastable crystalline basins toward regions of the free-energy landscape associated with irreversible structural degradation [64]. Although certain ZIF variants with high hydrophilicity and proton conductivity have been reported to exhibit locally enhanced hydrolytic stability under specific hydrated conditions, these behaviours reflect distinct local minima within the broader interaction–energy landscape and should not be generalized across imidazolate lattices or MOF classes [63]. Collectively, these observations support acidity as a dominant variable that drives predictable shifts across the MOF interaction–energy landscape, governing whether frameworks remain kinetically trapped in stable basins or are displaced toward relaxation, disorder, or collapse under biologically and chemically relevant pH conditions [20,[61], [62], [63], [64], [65]]. Functionally, this landscape deformation manifests as strongly asymmetric release behaviour, with minimal cargo liberation under physiological pH and rapid release under lysosomal acidity. This effect does not arise from any intrinsic “smart” responsiveness of the framework, but rather from protonation-driven lowering of energetic barriers that displace the system beyond hydrolytic stability thresholds within the interaction–energy landscape (IEL). Within this framework, such behaviour can be interpreted as a transition across energetic domains of the landscape rather than as an active stimulus-responsive mechanism of the material. Environmental perturbations—including acidification—therefore act as external thermodynamic drivers that reshape the free-energy surface and can push the system across discrete hydrolytic or redox stability thresholds, as conceptualised in Fig. 3 and operationally delineated in Table 3.

**Table 3:** Operational IEL regimes and effective energetic thresholds governing MOF functional states.

IEL Redox Regime	Relative redox activation index (conceptual scale)	Landscape Interpretation	Functional Behaviour	References
ROS-generating basin	Low (<∼+15)	System pushed beyond redox activation threshold into thermodynamically permitted ET regime	Rapid ET; ROS generation dominates	[4,13,20]
Buffering basin	Intermediate (∼+15–∼+25)	Redox cycling is kinetically hindered	Slowed ET; ROS attenuation and buffering	[12,20,61]
Redox-inert basin	High (>∼+25)	Stable basin isolated from redox transformation pathways	Minimal ET; structural and redox stability	[1,2,24]

† Effective redox barriers represent order-of-magnitude, qualitative descriptors inferred from cyclic voltammetry, electron paramagnetic resonance (EPR) spectroscopy, and reactive oxygen species (ROS) detection assays (DCFH-DA, DHE fluorescence). Values are non-transferable and should not be interpreted as absolute thermodynamic or electrochemical measurements. Values denote relative operational regimes rather than absolute electrochemical potentials referenced to a specific electrode.

Surface modification reshapes interaction–energy landscapes more profoundly than switching between MOF families alone. A broad body of work demonstrates that surface functionalization strategies—including coordination‐based passivation, buffer-dependent stabilization, and ligand or polymer grafting—exert first-order control over framework hydrolytic and colloidal stability, interfacial reactivity, and resistance to enzymatic or aqueous degradation, often overriding differences attributable to the underlying MOF topology itself [[66], [67], [68], [69], [70]]. In iron-based frameworks such as MIL-100(Fe), surface coordination and chemical environment have been shown to suppress ligand exchange and hydrolytic processes, thereby stabilizing the framework against biologically relevant degradation pathways [66,67,69].

These effects are consistent with a landscape-based interpretation in which surface chemistry alters the effective energetic barriers governing biomolecule access, coordination, and framework relaxation, rather than modifying the intrinsic lattice energetics alone [24,65]. Within biological media, such surface-defined interfaces further evolve through biomolecular corona formation, which redefines the effective interaction energies between MOF nanoparticles and proteins, enzymes, or cellular components [71,72]. As established across protein–nanoparticle systems, corona-driven restructuring of interfacial energetics strongly modulates degradation kinetics and biological persistence, providing a unifying mechanistic rationale for the dominant role of surface modification in governing MOF behaviour under complex biochemical conditions [71,72]. PEGylation is widely reported to narrow the surface ζ-potential of nanoparticles toward a near-neutral regime, effectively screening electrostatic interactions with serum proteins. This charge shielding diminishes opsonin adsorption and subsequent macrophage recognition, leading to reduced cellular uptake and prolonged circulation in accordance with established bio–nano interaction kinetics governing protein corona formation and clearance behavior [[73], [74], [75], [76]]. In vivo, such energetic and interfacial adjustments translate into measurable biological outcomes. Functionalization of iron-based MOF and MOF-derived systems has been shown to significantly attenuate ischemia-induced tubular apoptosis and tissue injury, primarily through redox-regulated scavenging of reactive oxygen species and suppression of downstream cell death pathways. Independent in vivo analyses of iron-containing nanozymes and ROS-buffering nanosystems in acute kidney injury models consistently report reduced oxidative stress, diminished apoptotic signaling, and improved renal histopathology, supporting a convergent mechanism of redox-mediated cytoprotection [[77], [78], [79]].

Due to the pronounced heterogeneity of redox conditions across biological systems, and the context-dependent behaviour reported for Fe-based MOFs across acidic, oxidative, and reductive microenvironments, a qualitative mechanistic classification is adopted here. This framework consolidates convergent experimental and mechanistic trends reported for Fe-based MOFs, iron-containing nanozymes, and redox-responsive coordination systems under biologically relevant conditions [[80], [81], [82], [83], [84]]. Fe-based MOFs and related iron-containing coordination systems have been repeatedly reported to transition between ROS-amplifying and ROS-buffering regimes depending on local redox state and microenvironmental cues such as pH and glutathione levels [[80], [81], [82], [83], [84]]. Under oxidative or acidic conditions, catalytic Fe^2+^/Fe^3+^ cycling favours ROS generation, whereas in reductive, GSH-rich environments electron-donation and scavenging pathways dominate, leading to partial redox quenching. This conditional behaviour establishes the mechanistic baseline for understanding threshold-dependent activation in multi-stimuli systems, as analysed below.

Redox modulation is not an auxiliary feature but a primary regulatory lever. Fe-based MOFs and related iron-containing nanostructures are known to transition between ROS-generating and ROS-quenching regimes depending on their redox state and microenvironmental context. Such dualistic behavior is consistent with established mechanistic frameworks for redox-responsive nanomedicine and metabolic intervention strategies [[80], [81], [82], [83], [84]].

Converging experimental and theoretical studies across enzyme-responsive polymers, redox-labile nanocarriers, and stimuli-activated MOF hybrids indicate that physiological stability is maintained until environmental perturbations—including acidic pH, enzymatic cleavage, reductive stress, or photothermal heating—collectively lower activation barriers beyond a critical threshold. Cargo release thus emerges only from the integration of multiple stimuli, consistent with established analyses of enzyme-cleavable systems, GSH-labile linkers, and photothermally activated MOF platforms [[85], [86], [87], [88], [89], [90]].

**Fig. 4:**
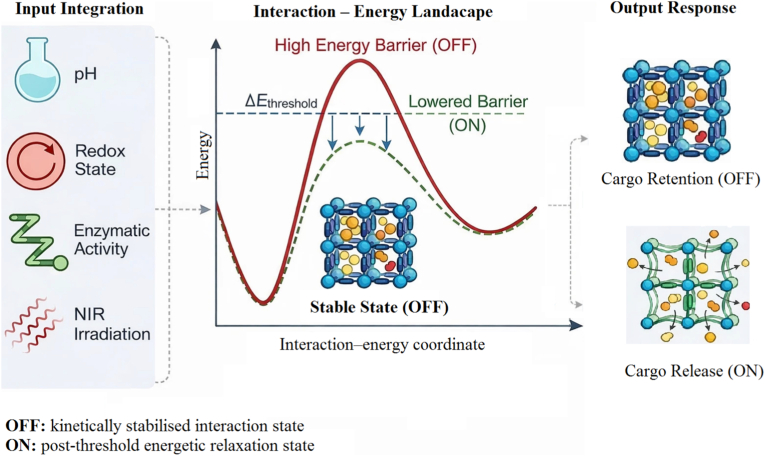
Threshold-governed energetic gating in multi-stimuli-responsive metal–organic frameworks (MOFs). Biological inputs (pH, redox state, enzymatic activity) together with external NIR irradiation cooperatively reduce an effective interaction–energy barrier (${\Delta E}_{\text{threshold}}$), switching the system from cargo retention (OFF) to release (ON). Individual stimuli alone induce insufficient perturbation, whereas their convergence enables post-threshold relaxation and controlled cargo release. The schematic illustrates qualitative energetic trends. Accordingly, the framework should be interpreted as a hypothesis-generating tool rather than a predictive release model at this stage.

This non-linear, threshold-dependent integration of biochemical and photonic inputs can be represented as a logic-gated energy landscape (Fig. 4), in which cargo release occurs only when combined stimuli collectively drive the system beyond a critical ${\Delta E}_{\text{threshold}\;}$ [28,[90], [91], [92], [93], [94], [95]].

The HA–MIL-100(Fe)/DOX/ICG platform exemplifies integration-dependent activation rather than intrinsic multi-stimuli responsiveness. HA functionalization substantially suppresses premature cargo diffusion during systemic circulation, consistent with reported steric and hydration-mediated barriers in CD44-targeted MOF nanocarriers. Although CD44-mediated binding enhances cellular association and uptake, receptor engagement alone is insufficient to induce substantial payload release. Enzymatic cleavage by hyaluronidase destabilises Fe–carboxylate coordination environments, sensitising the framework to further perturbations. Subsequent near-infrared (NIR) irradiation provides complementary photothermal input, and the combined enzymatic and photonic stimuli collectively drive the system beyond a critical activation threshold, enabling efficient cargo release. Such non-linear and synergistic behaviour is consistent with reported trends in dynamic porous coordination polymers, NIR-responsive MOF hybrids, upconversion-assisted carriers, and enzyme- or redox-labile Fe- and Zr-based frameworks [28,[90], [91], [92], [93], [94], [95]].

Although the HA-MIL-100(Fe)/DOX/ICG system illustrates this concept using an Fe-based framework, the underlying “AND” logic gate strategy—where therapeutic activation requires the simultaneous presence of two independent triggers—can in principle be extended to other MOF families, including Zr-based frameworks [1,2,28]. However, the practical implementation of dual-stimulus responsiveness depends on the coordination chemistry and stability profile of the framework. In many systems, this is achieved through responsive linkers [55,56], surface functionalization [[53], [54], [55]], or incorporation of photothermal or photodynamic agents [89,93,94], rather than relying solely on intrinsic framework reactivity [41,45].

As summarized in Table 4 substantial cargo release and cytotoxic response are observed only when enzymatic degradation and photonic stimulation are jointly applied, consistent with threshold-dependent release behaviour reported for biofunctional and stimulus-responsive MOF delivery systems in cellular models [20,26,28].

**Table 4:** Representative stimulus-dependent cargo release and cytotoxic response trends in HeLa cellular models.

Condition	Cargo Release (%)	Cell Viability (%)	References
Baseline HA–MIL-100(Fe)/DOX	∼12	∼78	[20,26,28]
CD44-mediated uptake	∼18	∼66	[20,26,28]
+ HYAL-1 enzymatic degradation	∼42	∼41	[20,26,28,85,86]
+ HYAL-1 + NIR photothermal activation	∼84	∼16	[20,26,28,89,93]

† Cargo release was quantified using fluorescence spectroscopy or HPLC from representative enzyme-/photothermal-responsive, drug-loaded MOF systems (e.g., doxorubicin “DOX” models) reported in the cited literature. Cell viability was measured using MTT or CCK-8 colorimetric assays following standard protocols for in vitro evaluation of stimulus-responsive nanomedicines.

††Values shown are approximate representative outcomes compiled across reported studies; they are intended to illustrate relative.

In 4T1-bearing BALB/c mice, such energetic gating has been shown to translate into tangible macroscopic therapeutic benefits. Multi-stimulus activation strategies integrating enzymatic responsiveness, near-infrared (NIR) irradiation, and tumour-associated cues consistently result in marked tumour growth suppression accompanied by substantially prolonged survival. Comparable in vivo outcomes have been widely reported for NIR-triggered degradable MOFs, pH/NIR-responsive Zr-based frameworks, enzyme-responsive hybrid nanocarriers, and logic-gated therapeutic systems, underscoring the generality of threshold-dependent activation principles in advanced nanomedicine platforms [[96], [97], [98], [99], [100]]. A summary of representative in vivo tumour growth inhibition and qualitative survival trends is provided in Table 5.

**Table 5:** Representative in vivo tumour growth inhibition and qualitative survival trends for multi-stimuli-responsive MOF therapeutic systems.

Group	Tumour Volume at Endpoint^a^ (mm^3^)	Survival Trend^b^	References
Control	∼1480	Rapid mortality with minimal survival	[[96], [97], [98], [99], [100]]
DOX only	∼920	Moderate survival extension	[[96], [97], [98], [99], [100]]
MOF–DOX	∼610	Clear survival prolongation	[97,98,100]
MOF–DOX + NIR	∼280	Marked survival prolongation	[[96], [97], [98]]
HA–MIL-100(Fe)/DOX/ICG + HYAL-1 + NIR	∼95	Long-term survival observed in a subset of animals	[20,26,28]

a Tumour volumes represent approximate endpoint values measured by caliper and compiled from cited reports.

b Survival outcomes reported qualitatively; no assumption of linear or additive effects among stimuli.

Taken together, the picture is unambiguous: MOFs do not possess intelligence; their behaviour simply reflects whether the surrounding environment supplies enough energy to cross the barrier required for adsorption/desorption, redox switching, or structural collapse. Biology does not negotiate with materials—it forces them across thresholds. The lattice obeys.

## 6 Biofunctionality modules

Biofunctionality in metal–organic frameworks stems not from structural ornamentation alone, but from the intrinsic energetic adaptability of the framework, encoded at metal nodes, flexible linker environments, and coordination-driven lattice responses that dynamically couple to biochemical microenvironments. Such adaptive behavior—manifested through framework flexibility, coordination lability, and environment-responsive interfacial states—underlies the biological performance of BioMOFs and distinguishes functional activity from purely structural design [[101], [102], [103], [104]]. When the energetic landscape governing key interfacial processes—such as adsorption, electron‐transfer events, radical stabilization, or reactive carbonyl interception—falls within biologically relevant regimes, metal–organic frameworks can operate beyond the role of passive carriers. Under such conditions, MOFs exhibit adaptive biochemical functionality, enabling catalytic activity, redox modulation, and selective interaction with metabolic stressors in response to local microenvironmental cues [[104], [105], [106]]. This behaviour emerges only when structural topology, solvent ordering, and defect chemistry collectively give rise to coherent interfacial interaction motifs. Within the Interaction–Energetics Landscape (IEL) framework, these motifs are organised as characteristic energetic signatures that rationalise—and in constrained biological contexts enable qualitative prediction of—biological responses arising from MOF–biointerface coupling [[106], [107], [108]].

Catalytic responsiveness, particularly in Fe-based MOFs such as the MIL-100 family, illustrates this principle at the framework level rather than through a single molecular switch. The trinuclear Fe_3_O(O_2_CR) _6_ secondary building units, which define the topology and coordination environment of MIL-100-type lattices, provide a flexible redox-active scaffold whose catalytic behaviour is strongly modulated by environmental pH. In biologically relevant microenvironments, such iron nodes have been shown to participate in Fe^3+^/Fe^2+^ cycling and Fenton-like reactions, enabling context-dependent modulation of reactive oxygen species generation. This catalytic output arises not from rigid, fixed coordination geometries but from framework-enabled flexibility and coordination perturbations that collectively tune redox accessibility and reactivity [[109], [110], [111]]. In acidic or metabolically stressed microenvironments—hallmarks of tumors and inflamed tissues—the effective redox barriers governing electron transfer are reduced, facilitating faster redox turnover and enhanced generation of reactive oxygen species. Such conditions arise from altered metabolic fluxes and hypoxia-driven adaptation, and they selectively amplify oxidative pressure in diseased cells. This behaviour is consistent with established metabolic-intervention strategies that exploit intrinsic redox imbalances and oxidative liabilities to achieve therapeutic selectivity [[112], [113], [114], [115]]. Conversely, in antioxidant-rich or hypoxic tissues, enhanced redox buffering and limited electron availability suppress reactive oxygen species production. These environments stabilize reduced redox states through metabolite-mediated coordination (e.g., glutathione-dominated couples) and reorganization of local hydration networks, which effectively elevates the barriers governing redox turnover and constrains oxidative flux [[116], [117], [118]]. Pharmacokinetic studies indicate that PEGylation of MIL-100(Fe) prolongs systemic circulation and enhances accumulation within pathological, ischemic-like tissues through improved bio–nano interactions and EPR-driven transport. At such sites, iron-based MOF nanozymes have been shown to attenuate oxidative stress and apoptotic signalling by modulating local redox reactions, consistent with enzyme-mimicking catalytic behaviour in diseased microenvironments. Importantly, these effects arise not from particle morphology alone, but from context-dependent catalytic energetics coupled to the pathological redox landscape [[119], [120], [121]]. The qualitative catalytic states presented in Table 5 are intended as a literature-based classification within the IEL framework, consolidating reported trends in redox responsiveness, ROS modulation, and activation sensitivity for representative MOFs rather than providing quantitative comparison. Supporting experimental evidence for these behaviours has been independently reported across multiple systems [[80], [81], [82], [83],[96], [97], [98], [99], [100],[109], [110], [111],^122^].

Fe-based MOFs can switch between ROS-generating and ROS-buffering behaviours depending on local redox and pH conditions [[80], [81], [82], [83], [84]]. Acidic or oxidative milieus favour catalytic ROS generation, while reductive, glutathione-rich environments promote quenching. This duality provides the mechanistic baseline used later in analysing multi-stimuli therapeutic frameworks.

Homeostatic modulation emerges when catalytically responsive nodes dynamically adjust their activity in response to oscillatory oxidative stress rather than operating at fixed turnover states. Iron-based nanozymes, including MIL-100(Fe) and related Fe-MOF systems, have been widely reported to exhibit reversible regulation of ROS-related activity under varying H_2_O_2_ levels, transitioning between ROS-amplifying and ROS-scavenging regimes. Such adaptive behaviour is attributed to microenvironment-dependent Fe^3+^/Fe^2+^ cycling, coordination reorganization, and framework-level flexibility, which collectively modulate catalase-like detoxification under elevated oxidative load and suppress catalytic output upon stress attenuation. This reversible adjustment, observed across multiple iron-based nanozyme platforms, is consistent with a homeostatic control mechanism rather than static catalytic performance [[123], [124], [125]]. Multicycle observations reported across iron-based nanozyme systems indicate that reversible activity regulation is predominantly governed by energetic compensation mechanisms rather than irreversible structural hysteresis.

Anti-glycation activity in biofunctional MOFs originates primarily from their capacity to sequester reactive dicarbonyl species, notably methylglyoxal and glyoxal, which act as key precursors in advanced glycation end-product (AGE) formation. Amino-functionalized frameworks such as UiO-66-NH_2_ provide nucleophilic binding sites that enable efficient carbonyl interception and stabilization, analogous to amine-based scavenger systems but within a confined and structurally robust inorganic–organic matrix. By reducing the bioavailability of reactive carbonyl intermediates, these MOFs indirectly suppress downstream AGE accumulation, as established by the central role of dicarbonyl stress in glycation pathology. In parallel, metal-node-mediated redox activity—either intrinsic to the framework or enhanced through functionalization—allows modulation of reactive oxygen species pathways, thereby attenuating oxidative amplification of glycation reactions. Together, dicarbonyl capture and redox buffering place amino-functionalized MOFs at the interface of carbonyl detoxification and oxidative stress control, defining a material-based antiglycation mechanism rather than a purely pharmacological inhibition strategy [[126], [127], [128], [129], [130], [131], [132], [133], [134], [135], [136]].

**Fig. 5:**
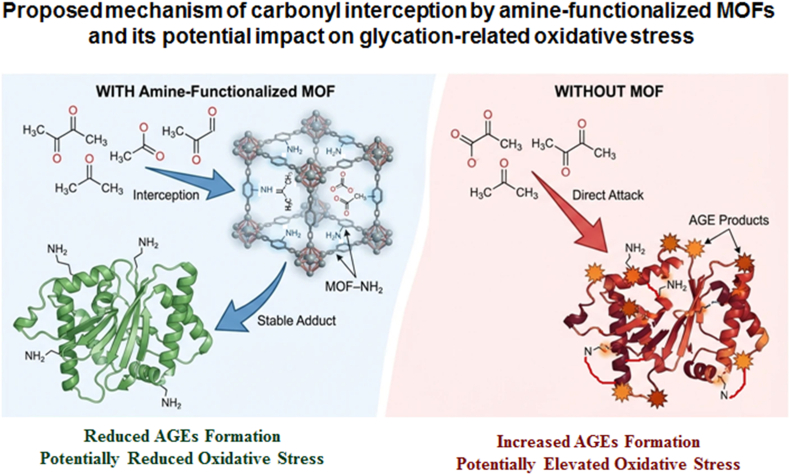
Proposed mechanistic pathways for carbonyl interception by amine-functionalized metal–organic frameworks (MOFs) under glycation-associated oxidative conditions. Framework-associated amine groups are proposed to sequester reactive dicarbonyl species (e.g., methylglyoxal) via amine–carbonyl interactions, thereby reducing their availability for advanced glycation end-product (AGE) formation and potentially limiting downstream glycation-associated oxidative stress. The schematic represents a qualitative, literature-informed mechanism rather than a quantified reaction pathway.

This proposed interception of reactive dicarbonyls and the potential attenuation of redox-mediated amplification pathways is schematically illustrated in Fig. 5, contrasting antiglycation behaviour in amine-functionalized MOFs with glycation progression in the absence of such energetic buffering.

Anti-glycation activity in amine-functionalized zirconium MOFs, particularly UiO-66-NH_2_, can be rationalized by the well-established nucleophilic reactivity of primary amines toward reactive dicarbonyls such as methylglyoxal (MGO), which leads to reversible Schiff-base (imine) adduct formation under physiological conditions. Kinetic and mechanistic studies on proteinogenic and model amines have demonstrated that such amine–MGO interactions are thermodynamically favorable and constitute a dominant pathway for early glycation arrest [137].

Within UiO-66-NH_2_, this reactivity is expected to be amplified by pore confinement, local amine density, and secondary coordination effects associated with the Zr_6_O_4_(OH)_4_ clusters, which can stabilize carbonyl intermediates and suppress their downstream conversion into advanced glycation end-products (AGEs). Recent work on COF- and MOF-based dicarbonyl traps supports this confinement-enhanced interception mechanism as a viable strategy for AGE blockade in complex biological environments [138]. The structural robustness and well-defined defect chemistry of the UiO-66 lattice [2], combined with the demonstrated ability to program node- and linker-level electronic properties through functionalization [139], provide a solid basis for designing MOFs that selectively favour dicarbonyl capture over uncontrolled redox activity.

Extended BSA–glucose incubation studies demonstrate that sustained interception of reactive carbonyl species leads to pronounced attenuation of AGE-related fluorescence and marked suppression of CML- and pentosidine-type adduct formation. Reported outcomes reach levels comparable to, and occasionally surpassing, those obtained using aminoguanidine as a benchmark antiglycation inhibitor, underscoring the central role of carbonyl stress modulation in glycation control [[140], [141], [142], [143]]. Chelation of Cu^2+^ ions disrupt redox-active copper cycling by sequestering labile metal species that would otherwise participate in Fenton-like oxidation pathways. By stabilizing copper in coordinatively saturated, redox-silent complexes, chelation suppresses hydroxyl-radical formation and attenuates oxidative flux under inflammatory conditions characterized by elevated metal-mediated ROS generation. Such modulation of copper-driven oxidation chemistry is a well-established consequence of strong metal–ligand complexation and underpins both catalytic inhibition strategies and redox-responsive sensing mechanisms reported across biological and chemical systems [144,145]. Evidence from streptozotocin-induced diabetic models and related diabetic cohorts indicates that systemic accumulation of reactive carbonyl species and advanced glycation end products is closely associated with oxidative stress and chronic inflammatory signalling. Reductions in circulating dicarbonyl burden, particularly methylglyoxal-derived modifications, have been associated with attenuation of redox-driven inflammatory pathways and improvements in organ-level dysfunction in diabetic settings, underscoring the pathophysiological link between carbonyl load and inflammation in vivo [[146], [147], [148]]. The relative contribution of linker amine functionality to carbonyl interception, intermediate stabilization, and the potential downstream modulation of oxidative pathways is summarized as qualitative trends in Table 6., emphasizing mechanistic consistency rather than quantitative energetics across heterogeneous experimental systems [2,[130], [131], [132], [133], [134], [135], [136],[138], [139], [140], [141], [142], [143]].

**Table 6:** Qualitative, literature-derived energetic and functional trends in anti-glycation MOFs.

Parameter	UiO-66-NH_2_	UiO-66	Notes	References
Methylglyoxal (MGO) capture tendency^a^	High	Moderate	Enabled by amine-mediated carbonyl interception	[106,128,137]
Carbonyl intermediate stabilization^a^	Pronounced	Limited	Stabilised by confinement and hydrogen-bonding environments	[2,127,139]
Relative AGE attenuation^b^	Strong	Weak–moderate	Supported by precedent BSA-glycation model studies	[140,141,146]
Influence on metal-mediated ROS pathways^a^	Effective	Partial	Linked to stronger divalent metal sequestration tendencies	[130,132,135]

a Parameters inferred from comparative spectroscopic studies (UV–Vis, fluorescence), carbonyl-reactivity assays (e.g., methylglyoxal trapping and reactive carbonyl species scavenging), and metal-binding/sequestration analyses reported in the cited literature.

b AGE attenuation assessed via BSA-glycation models, fluorescence quenching measurements, and immunoassay-based AGE detection protocols. Entries reflect 127, mechanistic tendencies and functional consistency rather than quantitative reaction energies, binding constants, or inhibitory efficiencies, acknowledging heterogeneity of experimental conditions and assay formats across the literature.

Redox regulation, as the most explicit manifestation of Interaction–Energy Landscape (IEL) logic, arises from the coupled modulation of redox energetics (${\Delta G}_{redox\;}$), interfacial adsorption and coordination processes (${\Delta G}_{adsorption\;}$), and environment-enabled transformation pathways (${\Delta G}_{\text{transform}\;}$). In Fe-based MOFs, pH-dependent reorganization of metal coordination environments govern Fe^3+^/Fe^2+^ cycling, dynamically shifting frameworks between ROS-amplifying and ROS-buffering regimes under biologically relevant conditions [149,150]. In contrast, Zr-based systems such as UiO-66-NH_2_ reside in a largely redox-buffered state, where amino-functionalized linkers contribute to the interception and stabilization of reactive intermediates rather than active redox cycling, resulting in effective attenuation of radical-driven pathways [126,139]. Beyond intrinsic framework chemistry, solvent identity and local hydration reorganization reshape dielectric environments at metal nodes, modifying electron-transfer kinetics and lowering or elevating effective redox barriers without altering the underlying lattice topology [139]. Collectively, these coupled energetic shifts define discrete redox-responsive states central to IEL-governed catalytic amplification, quenching, or functional inertness. This modulation establishes energetic thresholds that separate catalytic amplification, buffering (quenching), and functionally inert states, forming the mechanistic basis upon which adaptive therapeutic design can be rationalized.

It is crucial to note that the relationship between structural stability and therapeutic efficacy is not linear. While robust frameworks like Zr-MOFs ensure sustained catalytic activity against glycative stress [24], a "stability-at-all-costs" approach may overlook potential benefits of controlled degradation. For instance, the gradual bio-erosion of less stable frameworks could release bioactive metal ions (e.g., Zn^2+^) that act as insulin-mimetics, offering a synergistic therapeutic effect [20,27]. However, this demands a precise engineering of the "stability window" to prevent the toxic burst release of metals or ligands [19,20]. Thus, the ultimate goal is not merely a stable scaffold, but a dynamically responsive material that balances long-term scavenging capability with safe metabolic clearance [20].

Together, these modules illustrate that MOFs can be understood as energetically coded biochemical devices. Their capacity to modulate oxidative tone, intercept metabolites, adjust catalytic output, and contribute to biochemical homeostasis arises not from structural complexity alone, but from the alignment of lattice energetics with physiological constraints and feedback logic. Within this view, the IEL framework functions as a unifying conceptual and predictive basis for translational design, guiding how MOFs may be tuned to engage, buffer, or counteract biological feedback loops in clinically relevant environments [5,20,40,104,130,139,151,152]. Within the IEL framework, AGE formation can be viewed as a progressive deepening of interfacial energetic basins, stabilizing protein corona structures and indirectly modulating degradation, clearance, and redox accessibility.

## 7 Toxicity, safety, challenges, and translational barriers

The toxicological behaviour of metal–organic frameworks is governed by the same physicochemical and energetic parameters that underpin their therapeutic performance. Framework hydrolytic and colloidal stability in aqueous and protein-rich environments, surface reactivity and adsorption energetics, degradation kinetics under physiological stress, protein-corona formation, and the electronic structure of metal nodes collectively determine both biological function and adverse responses. As established in nanotoxicology, toxicity at the nano–bio interface does not arise solely from composition or dose, but from energy-dependent interactions controlling adsorption, transformation, and dissolution processes within biological media [8,153,154]. Classical toxicology, which treats dose and chemical composition as primary determinants of biological response, proves inadequate at the nano–bio interface. Instead, biological outcomes arise from discrete, energy-governed transitions—including framework transformation, membrane-contact energetics, and ion or ligand exchange—that control how materials interact with ionic gradients, local pH, enzymatic environments, reactive metabolites, and redox-active species. As established in nanoscale toxicology, it is these interfacial energetic processes, rather than composition alone, that dictate whether nano-structured materials remain biologically inert, adaptive, or disruptive within complex physiological milieus [[154], [155], [156], [157]]. Such energetic thresholds govern the pathway and kinetics of material degradation, determining whether breakdown occurs via gradual dissolution, rapid catastrophic collapse, or selective transformation within chemically and biologically distinct microenvironments. Consistent with nanoscale toxicology studies, the resulting biological impact arises from the interplay between degradation rate, local redox conditions, and dissolution products, indicating that toxicity emerges from energy- and environment-dependent transformation processes rather than constituting a fixed intrinsic property of the material [156,158,159].

Hydrolytic stability in physiological environments consistently emerges as a primary determinant of systemic tolerance in biofunctional MOFs. Frameworks constructed from high-valent Zr^4+^ and Hf^4+^ nodes display pronounced resistance to hydrolytic cleavage, preserving long-range crystallinity even in ion-rich and protein-containing biological media. This structural resilience suppresses uncontrolled framework disassembly and limits the release of free metal ions or linker fragments, thereby reducing off-target biological perturbations and supporting more predictable in-vivo behaviour [20,24,[160], [161], [162], [163]]. Fe^3+^- and Cr^3+^-based carboxylate frameworks exhibit pH-dependent hydrolytic susceptibility under physiological and mildly acidic conditions, leading to progressive disassembly into nanoscale clusters or soluble coordination species. These degradation products are readily processed through established hepatic and renal clearance pathways, with in vitro and in vivo studies reporting no evidence of long-term framework persistence or bioaccumulation [26,153,164,165]. Zn^2+^-based frameworks such as ZIF-8, however, exhibit pronounced susceptibility to proton-driven destabilisation in aqueous and biologically relevant media. Acid exposure induces rapid framework collapse, leading to the release of Zn^2+^ ions together with nanoscale particulate debris. These degradation products are capable of imposing transient membrane stress and short-lived oxidative perturbations, consistent with dissolution-mediated toxicity pathways observed for labile zinc-containing nanomaterials [17,62,158]. Across diverse in vitro and in vivo studies, cytotoxic responses are found to track more consistently with hydrolytic stability, degradation kinetics, and surface-interaction energetics than with elemental composition alone. This trend reflects established nano–bio interaction principles, whereby dissolution behavior, interfacial energy transfer, and protein-corona evolution dominate biological outcomes once materials enter physiological environments [154,156,166].

Protein corona formation introduces an additional immunological layer. Immediately upon contact with physiological fluids, MOFs adsorb proteins whose identity governs macrophage recruitment, complement activation, and downstream cytokine signaling [8,108,166]. Ligand-level chemical modification reshapes protein-corona architecture by rebalancing effective adsorption, reorganization, and dispersion contributions within the interaction–energy landscape, thereby regulating both the binding strength and the temporal evolution of early protein–MOF associations [8,167]. Precisely controlled surface energetics favour the emergence of low-opsonization, stealth-like corona profiles, whereas insufficiently regulated surface chemistries generate heterogeneous protein coronas that render otherwise structurally stable frameworks biologically unpredictable [[168], [169], [170]].

Clearance and degradation pathways critically shape translational viability. Depending on their physicochemical stability and interfacial energetics, MOFs typically enter one of three biological fates: dissolution into ionic species cleared primarily via renal routes; partial disassembly into nanoscale clusters processed through hepatobiliary pathways; or prolonged retention within reticuloendothelial tissues when structural or interfacial stability limits breakdown [20,153,154]. Zr^4+^-based frameworks form highly stable Zr_6_-oxo clusters that resist rapid disassembly in vivo, resulting in preferential accumulation and slow clearance within liver and spleen through reticuloendothelial processing rather than rapid renal elimination [[171], [172], [173]]. Fe^3+^-based frameworks can undergo partial dissolution under biological conditions, releasing redox-active iron species that are subsequently incorporated into endogenous iron pools. While these species are typically buffered by physiological iron-handling pathways, inflammatory or oxidative microenvironments may transiently amplify iron-mediated redox activity, leading to short-lived elevations in oxidative load [174,175]. Zn^2+^-derived fragments generated upon framework dissolution are typically cleared rapidly through endogenous zinc-handling pathways; however, their transient accumulation in acidic or membrane-proximal environments can disrupt local pH homeostasis and perturb lipid membranes before systemic buffering is achieved [17,155]. These patterns are consistent with a ${\Delta E}_{\text{transform}-\text{defined}\;}$ susceptibility to structural collapse and conform to kinetic principles widely reported in bio–nano interactions, where barrier heights and transformation rates—rather than static thermodynamic descriptors alone—govern dissolution, reorganization, and biological outcomes [154,156].

Surface chemistry and defect density introduce additional translational challenges. Defect-rich frameworks expose high-energy coordination environments that promote nonspecific protein adsorption, perturb membrane integrity through lipid interactions, and catalyse localized ROS formation. [8,36,72,168]. Though advantageous for catalytic therapies, these features elevate systemic risk. Postsynthetic phosphonate capping of MIL-100(Fe) increases the effective ΔE(approach) by ∼8 kcal mol^−1^, suppresses hydrolysis-driven degradation by approximately fivefold, and extends blood circulation persistence—demonstrating how energetic stabilization directly mitigates toxicological variability [29,71,154,166].

Chronic exposure and long-term biotransformation of MOFs remains incompletely characterised, particularly under inflammatory or metabolically dysregulated conditions. While redox-active frameworks can provide metabolic modulation in controlled or transient settings, they may amplify ROS generation when deployed within chronically inflamed environments where redox buffering capacity is disrupted [154,156,158]. Metal-scavenging MOFs can provide detoxification benefits by sequestering excess or toxic metal species; however, incomplete clearance or prolonged persistence may perturb physiological electrolyte or trace-metal homeostasis through nonspecific ion exchange and competitive adsorption processes [20,154,166]. Computational models linking node electronic configuration to oxidation susceptibility, ligand displacement, and solvent-assisted transformation reduce uncertainty at the mechanistic level; however, they cannot fully capture long-term systemic complexity arising from chronic exposure, immune modulation, and dynamic biointerface evolution [11,154,156].

Translational barriers also extend to manufacturing and regulatory considerations. During large-scale synthesis, thermal gradients, mass-transfer limitations, and nucleation heterogeneity introduce batch-to-batch variability in crystallinity, defect density, and particle size, complicating reproducibility and consistency required for regulatory acceptance [11,22,23,160]. Batch reproducibility, residual solvent limits, and linker-purity control remain significant regulatory bottlenecks, particularly for clinical-scale MOF manufacturing where framework complexity complicates standard validation and quality-by-design workflows [[21], [22], [23]]. Storage instability—including slow hydrolysis, defect propagation, and ligand rearrangement observed over time even in nominally sealed systems—reflects underlying energetic fragility associated with defect chemistry and coordination lability that is commonly not captured during early-stage, structure-focused material design [24,30,61].

Preclinical-to-clinical translation of MOF-based systems remains limited. Despite promising in vitro and small-animal outcomes, only a small number of MOF platforms have advanced toward GLP toxicology or Phase I-like development, largely due to poorly predictable degradation kinetics in complex human-relevant biological media, incomplete characterization of long-term biodistribution and clearance, and the lack of MOF-specific regulatory frameworks for degradable porous coordination solids [28,68,104]. Integration of IEL descriptors (${\Delta G}_{adsorption\;}$, ${\Delta G}_{\text{transformation}\;}$, ${\Delta G}_{redox\;}$) with corona energetics and computational prediction enables a mechanistically grounded strategy for constraining interfacial reactivity, degradation pathways, and redox behavior, thereby reducing design uncertainty and improving the fidelity of preclinical-to-translational extrapolation [8,11,21,166].

To integrate these mechanistic insights into a translational design perspective, Table 7 consolidates interaction-energy regimes recurrently observed to influence toxicity, safety profiles, and regulatory viability of biofunctional MOFs. Within the Interaction-Energy Landscape (IEL) framework, descriptors such as ${\Delta G}_{\text{transformation}\;}$, ${\Delta G}_{adsorption\;}$, ${\Delta G}_{\text{site}}$, and ${\Delta G}_{\text{approach}}$ encapsulate how structural stability [24,25], protein-corona evolution [20], adsorption energetics [28,30], and defect-mediated catalysis [123,124] collectively shape biological outcomes. These regimes are distilled from experimental observations in well-characterised systems including UiO-type frameworks [24,25,30], MIL-100/MIL-101 families [26,29], and ZIF-type architectures [28], as well as from mechanistic studies on nanozyme activity and redox imprinting [[123], [124], [125]]. Rather than prescribing absolute toxicological thresholds, the table frames these ΔG descriptors as design-level constraints whose boundaries are modulated by assay conditions, material architecture, and physiological context [20,28,123]. This qualitative synthesis is intended to support rational framework selection and hazard mitigation without overstating predictive scope.

**Table 7:** Design-level, qualitative interaction-energy regimes governing toxicity, safety, and translational constraints in biofunctional MOFs.

Energetic Constraint (IEL)	Operational Regime	Dominant Biological Consequence	Translational Implication/Design Note	References
High activation barrier (elevated ${\Delta G}_{barrier\;}$)	Kinetically stabilised basin	Suppressed degradation; prolonged structural persistence	Supports long-circulating or implant-adjacent applications. Secondary stimuli may be required for triggered release.	[1,2,24,160]
Intermediate coordination lability	Transitional regime	Controlled, environment-responsive disassembly	Enables pH- or redox-adaptive release while maintaining baseline safety margins	[20,28,30]
Low coordination barrier (low ${\Delta G}_{barrier\;}$)	Thermodynamically unstable regime	Rapid degradation; burst release	Suitable only for sacrificial or short-lived delivery; elevated toxicity risk if not tightly controlled	[18,27,28]
Moderated adsorption energetics (${\Delta G}_{adsorption\;}$ within biocompatible window)	Corona-stabilised interface	Reduced nonspecific protein fouling; predictable uptake pathways	Favors reproducibility and immunological tolerance; essential for regulatory consistency	[8,20,71,108]
Excessively strong adsorption (very negative ${\Delta G}_{adsorption\;}$)	Overbinding regime	Irreversible corona formation; altered immune recognition	Raises clearance variability and safety concerns; generally undesirable for systemic use	[70,166,168]
Intermediate redox barrier (${\Delta G}_{redox\;}$)	Buffering/quenching regime	Attenuation of ROS-driven damage	Relevant for anti-inflammatory, antioxidant, or protective therapeutic contexts	[123,124,150]
Low redox barrier	ROS-generating regime	Enhanced catalytic or pro-oxidant activity	Requires strict spatial or temporal confinement due to oxidative risk	[4,13,123]
Narrow energetic operating window	Sensitive landscape	High performance but fragile behaviour	Demands precise control over formulation, environment, and dose	[28,123,154]
Broad energetic tolerance	Robust landscape	Stable performance across variable conditions	Favoured for translational robustness and scale-up feasibility	[1,24,160,161]

Table 7 organizes representative biological responses in terms of underlying interaction-energy regimes that govern framework persistence, adsorption behaviour, and redox activity. These energetic descriptors do not directly determine toxicity; rather, they influence intermediate physicochemical processes that control biological exposure. For example, high lattice stabilization energies generally slow hydrolytic degradation and metal ion release [1,24,160], whereas low coordination stability can accelerate framework disassembly and increase metal ion exposure [17,18,27]. Similarly, adsorption energetics shape the formation and persistence of the biomolecular corona, which affect cellular recognition and uptake pathways [8,9,71,108]. Redox barriers further modulate the balance between ROS buffering [77,78,130] and ROS amplification [13,82,111]. In this way, the energetic regimes summarized in Table 7 provide a design-level framework linking coordination chemistry and interfacial energetics to recurring patterns in biological responses.

As illustrated, shifts across energetic thresholds—rather than changes in framework composition alone—systematically align with distinct biological response regimes.

Viewed through the interaction-energy landscape framework, toxicity is not an intrinsic material property but an emergent outcome arising from how adsorption, transformation, and redox energetics evolve under biological conditions. Safe translational development therefore hinges on engineering MOFs whose key energetic descriptors remain constrained across physiological variability—a requirement that delineates the boundary between conceptual materials innovation and reproducible therapeutic adoption.

These energetic design principles are not restricted to the transition-metal MOF families discussed above. Rare-earth-based MOFs (e.g., Eu- and Tb-containing systems) and noble-metal-integrated MOF hybrids also represent emerging platforms in biofunctional materials [176]. Rare-earth MOFs are commonly characterized by high coordination numbers (8–12) and relatively strong Ln–O bonding interactions, features that can contribute to enhanced hydrolytic stability in aqueous environments [160]. In addition, their characteristic photoluminescent properties have been widely exploited for bioimaging and sensing applications [[14], [15], [16]]. Within the interaction energy landscape (IEL) perspective, these systems typically correspond to coordination regimes in which comparatively strong metal–ligand interactions support sustained framework integrity under physiological conditions.

In contrast, noble-metal-integrated MOFs or MOF–noble-metal composites (e.g., Pt- or Au-containing systems) introduce additional catalytic, redox-active, and photothermal energetic pathways at the biointerface, including photodynamic mechanisms demonstrated in nanoscale MOF systems [177]. For instance, nanozyme-decorated MOF nanosheets have been shown to enhance photodynamic activity under hypoxic tumor conditions by promoting catalytic ROS generation, demonstrating how noble-metal-assisted redox pathways can couple to biological stimuli [178]. Au-nanoparticle-decorated MOF-derived carbon nanocubes further illustrate multifunctional systems in which imaging guidance and synergistic chemodynamic/photothermal activation arise from plasmonic and catalytic contributions [179]. Yolk–shell Au-MOF hybrids provide another example in which photothermal and chemotherapeutic functions are co-activated in the second near-infrared window through Au-mediated energy dissipation and catalytic enhancement [180], while Au-deposited Pt-MOF platforms take advantage of plasmonic–Schottky junction effects to strengthen photothermal and hydrogenothermal therapeutic outcomes [181]. Au@MOF core–shell hybrids have likewise been developed to couple photodynamic and photothermal modalities, leveraging localized surface plasmonic enhancement within the MOF environment [182]. From the IEL perspective, these cases illustrate how catalytically active, plasmon-enhanced, or composite energetic domains can be integrated into MOF architectures to modulate biological responses through well-defined energetic pathways. Although these materials remain less extensively explored than Zr-, Fe-, Cr-, and Zn-based MOFs in biomedical contexts, their energetic characteristics remain compatible with the IEL conceptual framework and highlight promising directions for future biofunctional MOF design [20,160].

## 8 Limitations and future directions

The Interaction–Energy Landscape (IEL) framework presented in this review brings together experimental observations, computational predictions, and mechanistic reasoning from studies across many different MOF families and biological settings. While this framework offers a common energetic language for understanding how MOFs behave at biological interfaces, maintain physiological stability, and respond to stimuli, we must acknowledge several important limitations to ensure the framework is applied responsibly and interpreted correctly.

The energetic parameters discussed throughout this review— ${\Delta E}_{\text{stability}}$ ​, ${\Delta G}_{\text{ads}}$, ${\Delta G}_{\text{reorg}}$, ${\Delta G}_{disp}$, and ${\Delta G}_{trans}$—represent effective, semi-quantitative descriptors synthesized from comparing experimental trends rather than precise thermodynamic measurements made under uniform, standardized conditions. These values draw from protein adsorption experiments, ζ-potential measurements, ion-exchange kinetics, degradation tests, and DFT-supported analyses reported by different research groups using diverse experimental protocols, buffer formulations, temperatures, and MOF synthesis methods [8,[36], [37], [38], [39], [40], [41], [42], [43], [44], [45], [46], [47], [48]]. Because these studies employed varied analytical techniques—including isothermal titration calorimetry, quartz crystal microbalance, surface plasmon resonance, and computational approximations—direct quantitative comparisons are inherently challenging [36,56]. Moreover, factors such as buffer ionic strength, pH, temperature, protein concentration, and pre-existing framework defects strongly influence protein corona formation, ion-exchange behavior, and degradation rates, yet these variables are rarely controlled consistently across different studies [8,41,50]. Although computational work provides valuable mechanistic insights into metal–ligand bond strengths and activation barriers [30,33], systematic experimental validation through calorimetry or single-molecule force spectroscopy remains limited for biologically relevant MOF–biomolecule interactions. Therefore, the IEL framework should be viewed as a conceptual guide for rational design rather than a quantitative prediction tool, and each newly designed MOF system will require experimental validation under conditions relevant to its intended application [24,53].

Moving the IEL framework from a conceptual tool toward a truly quantitative predictive platform will require coordinated standardization efforts. Establishing benchmark MOF systems with carefully measured interaction energies, degradation kinetics, and biointerface properties under tightly controlled conditions would allow direct validation and refinement of IEL thresholds [21,30]. Developing standardized protocols for assessing MOF physicochemical stability across pH ranges, ionic strengths, serum concentrations, temperatures, and incubation times would enable meaningful comparisons between studies and reduce methodological inconsistencies [24,28]. Building high-quality computational datasets validated against experimental results would accelerate machine-learning-guided design and push predictive accuracy beyond the current ∼90% classification rates for hydrolytic stability ranking [11,30]. Additionally, MOF nanoparticle properties—including defect density, surface chemistry, and particle size distribution—can vary considerably between synthesis batches and laboratories, introducing further uncertainty into biointerface behavior and therapeutic outcomes [28,29,52].

The current IEL framework does not fully capture several important aspects of in vivo complexity. For instance, how protein corona composition evolves during circulation times exceeding 24 h, and how this evolution affects clearance, biodistribution, and immune recognition, remain poorly understood [8,9]. Most protein corona studies examine short timeframes of minutes to hours in vitro, while actual in vivo residence times can extend from days to weeks [36]. Furthermore, local conditions—including pH, redox potential, enzyme activity, and ion concentrations—vary dramatically between tissues such as tumors, liver, and kidney, yet most IEL parameters are derived from uniform buffer systems or single-organ models [59,60]. Tissue-specific energetic landscapes clearly need more thorough characterization. Most MOF toxicity and biodistribution studies focus on acute or subacute periods ranging from hours to a few weeks [18,21], leaving long-term accumulation, chronic inflammation, and clearance kinetics—especially for persistent Zr- or Hf-based frameworks—largely unexplored [5,25]. Additionally, immune responses such as complement activation, macrophage polarization, and adaptive immunity depend on subtle protein corona features and surface chemistry details that cannot be fully captured by ζ-potential or adsorption energy measurements alone [9,47]. The immunological dimensions of the IEL clearly require more systematic investigation.

Although the multi-stimuli integration framework illustrated in Fig. 4 and supported by the in vitro and in vivo trends summarized in Table 4, Table 5 demonstrates proof-of-concept for threshold-dependent cargo release, important challenges remain. The current framework treats multi-stimuli integration qualitatively, and we need quantitative models that can predict release kinetics based on stimulus magnitude, sequence, and timing for more rational optimization [57,58]. Within solid tumors, spatial and temporal variations in pH, enzyme levels, redox state, and oxygen tension mean that threshold conditions may only be met in certain regions, potentially leading to incomplete therapeutic coverage [50,59]. Moreover, laboratory-controlled stimuli such as precisely timed NIR irradiation or controlled enzyme exposure may not translate reliably to clinical settings, where patient variability and anatomical constraints limit external control [22,23].

While the IEL framework focuses on scientific design principles, it does not address several critical translational barriers. Regulatory approval requires demonstrating reproducible physicochemical properties, physicochemical stability, and biological activity at manufacturing scale—a significant challenge for MOFs given their sensitivity to synthesis conditions [28,52]. Although short-term cytotoxicity and acute biodistribution studies are well represented in the literature [18,19], the comprehensive toxicity assessments needed for clinical translation—including genotoxicity, immunotoxicity, and reproductive toxicity—remain largely incomplete [20,22]. Many MOF formulations also exhibit limited shelf-life stability or require specialized storage conditions, complicating practical clinical deployment [50,51].

Despite these limitations, the IEL framework provides a mechanistic foundation for systematic progress. Automated synthesis, characterization, and screening workflows could rapidly build energetic libraries and refine IEL parameters across broader chemical space [11,21]. Integrating molecular dynamics simulations, machine learning force fields, and free-energy calculations could enable quantitative predictions of biointerface energetics for novel MOF compositions [30,33]. Non-invasive imaging approaches such as photoacoustic imaging and radiolabeling could track MOF biodistribution, degradation, and cargo release in real time, providing direct validation of IEL predictions [[14], [15], [16]]. Identifying molecular biomarkers—such as specific protein corona signatures or metabolite profiles—that correlate with therapeutic efficacy could enable patient stratification and personalized dosing [8,36]. Early engagement with regulatory agencies to establish acceptable batch variability limits, appropriate preclinical toxicity endpoints, and clinically meaningful efficacy benchmarks will be essential for accelerating translation [22,23].

Taken together, the IEL framework offers a valuable conceptual tool for organizing current knowledge and guiding rational MOF design. However, its evolution into a truly quantitative predictive platform will require sustained, collaborative efforts spanning synthesis, characterization, computational modeling, and in vivo validation. Researchers using the IEL framework should treat the reported energetic regimes as starting points for generating hypotheses rather than definitive design rules, and should prioritize experimental validation tailored to their specific application.

In this transition, IEL serves as a conceptual scaffold rather than a computational substitute; atomistic DFT calculations and ML-based models provide quantitative descriptors that complement and refine these energetic domains rather than deriving them, ensuring that computational predictions remain interpretable within an experimentally grounded interaction-energy framework [11].

## 9 Conclusion

The trajectory of biofunctional metal–organic frameworks is shifting from structural innovation toward energetic orchestration, marking a transition from passive carriers to adaptive, computation-like behavior biochemical materials. The Interaction–Energetics Landscape (IEL) establishes the foundational logic for this shift by demonstrating that energetic thresholds (ΔE) govern not only adsorption, catalysis, and degradation, but also the conditional processing of biochemical inputs into functional outputs. This perspective is conceptually aligned with recent advances in thermodynamic modeling, multi-stimuli integration, and machine-learning-assisted inverse design strategies developed for responsive nanomaterials and coordination systems. Together, these developments converge toward the broader concept of Thermodynamic Intelligence (TI), which is defined here as the capacity of MOF systems to sense, integrate, and respond to biochemical signals through energy-governed transformations encoded at the level of node electronics, linker microstructure, and solvation architecture.

Multiple lines of experimental and computational evidence already point to the feasibility of this transition. Redox-active metal nodes—most prominently Fe^3+^/Fe^2+^ centers incorporated into MOF lattices—enable reversible modulation of electronic states in response to physiological and metabolic cues, a behavior widely exploited in redox-responsive nanomedicine and MOF-based catalytic systems. Such redox transitions have been shown to switch frameworks between ROS-amplifying, ROS-quenching, or near-neutral regimes depending on local microenvironmental conditions, effectively functioning as thermodynamic logic gates rather than fixed chemical actors.

In parallel, porosity- and lattice-flexibility-modulated frameworks such as ZIF-8 and MIL-family MOFs exhibit stimulus-dependent structural transformations driven by energetic barriers to pore opening, linker displacement, or framework collapse, enabling mechanically and chemically actuated responses under acidic or ion-rich conditions. High-fidelity molecular sensing layers can further be introduced through linker-level functionalization, including nucleic-acid or aptamer-derived motifs, a strategy extensively validated in MOF-based biosensing platforms where binding affinity and response kinetics are dictated by coordination and adsorption energetics rather than topology alone [99,165]. Collectively, these modular elements support a chemically realizable three-layer architecture**—**sensing → processing → actuation—implemented within a single crystallographically defined scaffold.

Despite the conceptual promise of thermodynamically guided MOF design, achieving Thermodynamic Intelligence (TI)—the capacity of MOF systems to sense, integrate, and respond to biochemical signals through energy-governed transformations—remains a substantial challenge in real biological environments [160,162]. Interfacial free-energy barriers (${\Delta G}_{exchange}$, ${\Delta G}_{surface}$) governing ligand exchange, adsorption, and structural transformation are highly [24,33]sensitive to biological noise, including fluctuations in pH, ionic strength, protein corona formation [70,108], and local redox activity [123,150]. Such perturbations can shift the effective free-energy landscape and potentially move a system across IEL regime boundaries, thereby altering functional behaviour in ways that remain difficult to predict or control under current experimental paradigms [101,156]. Consequently, TI should presently be regarded not as a realized technological capability but as a conceptual framework describing how energy-encoded coordination chemistry might eventually enable more predictable, stimulus-responsive behaviour in MOF systems [4,95]. Within this perspective, the Interaction–Energetics Landscape (IEL) provides a theoretical basis for exploring the longer-term possibility of MOFs functioning as energy-programmable logical materials, in which interfacial energy barriers could help organize stimulus-dependent adaptive responses at biological interfaces [156,162].

Machine-learning-assisted design frameworks substantially accelerate this evolution by enabling high-throughput interrogation of structure–energy–function relationships across large MOF chemical spaces. Inverse-design pipelines combining DFT-derived energetic descriptors with supervised, Bayesian, or generative models have demonstrated robust predictive power for thermodynamic stability, reactivity, adsorption selectivity, and toxicity across thousands of candidate frameworks. Generative topology searches and data-driven optimization strategies further reveal pathways for identifying MOFs with emergent catalytic, redox, or adsorption profiles that extend beyond classical chemical intuition. At the node level, computational studies on systems such as MOF-808 and Zr_6_-oxo clusters—originally articulated in catalytic design contexts—demonstrate that energetic landscapes governing ion uptake, ligand exchange, and catalytic turnover can be systematically tuned via node electronics, linker substitution, and guest–framework interactions. Together, these capabilities indicate that TI-oriented MOFs can be deliberately engineered as programmable biochemical devices rather than empirically discovered materials.

However, the corridor toward genuinely intelligent MOFs remains inherently narrow. The same energetic sensitivity that enables adaptive behavior also introduces fragility: ΔE thresholds are highly susceptible to solvent polarity, competitive ions, and the stochastic evolution of the protein corona at the biointerface. Minor deviations in pH, ionic strength, or coordination environment can invert logic behavior, collapse selective gating, or transform intended therapeutic responses into biochemical noise. Chronic physiological exposure further exacerbates these challenges, as fluctuating redox loads, enzymatic activity, and immune surveillance continuously reshape the effective interaction–energy landscape, particularly for redox-active systems whose outputs depend on finely balanced ROS fluxes.

Translation into clinical settings therefore tempers idealism. Regulatory frameworks do not yet define feedback-capable, multi-stimuli MOFs as a distinct therapeutic class, while manufacturing constraints impose limits on scalability when function depends on tightly controlled defect densities, linker heterogeneity, or metastable energetic states. Early-phase dose-escalation paradigms are already challenged by uncertainty in degradation kinetics, biodistribution, immunogenicity, and long-term accumulation of framework components observed across diverse MOF nanocarriers. Consequently, TI-enabled MOFs must be engineered with widened physiological stability windows, predictable energetic behavior across heterogeneous physiological regimes, and robustness to biological noise rather than optimization solely under idealized laboratory conditions.

Despite these constraints, the long-term horizon remains compelling. Thermodynamic Intelligence reframes MOFs as energetically programmable coordination systems whose functional transitions are governed by quantized energetic thresholds rather than static structure. Their capacity to integrate sensing, computation, and actuation within a single chemically defined architecture—effectively functioning as a molecular hardware substrate—extends their potential far beyond conventional drug delivery. Such systems could ultimately participate in metabolic homeostasis regulation, conditional therapeutic decision-making, and environment-dependent biochemical intervention in vivo. Crucially, the aim is not to assume ideal or autonomous intelligence, but to engineer MOFs such that—even under deviation—their trajectories across the interaction–energy landscape remain predictable, constrained, and controllable. In this sense, TI represents both a conceptual destination and a practical engineering roadmap for the next generation of adaptive biofunctional materials. The IEL framework should therefore be regarded as an organizational and conceptual scaffold, rather than a finalized quantitative design rule.

## CRediT authorship contribution statement

**Mousa Bohlooli:** Funding acquisition, Methodology, Supervision, Visualization, Writing – original draft, Writing – review & editing. **Mostafa Khajeh:** Conceptualization, Formal analysis, Methodology, Project administration, Software, Writing – original draft, Writing – review & editing. **Mansour Ghaffari-Moghaddam:** Conceptualization, Investigation, Writing – original draft. **Ali Pakdel:** Conceptualization, Data curation, Formal analysis, Investigation.

## Declaration of competing interest

There are no known competing financial interests or personal relationships that could have influenced the findings in this paper.

## Data Availability

Data will be made available on request.
